# The genome sequence of the segmented worm, *Sthenelais limicola* (Ehlers, 1864)

**DOI:** 10.12688/wellcomeopenres.18856.1

**Published:** 2023-01-19

**Authors:** Teresa Darbyshire, Mitchell Brennan, Sean McTierney, Marine Biological Association Genome Acquisition Lab, Darwin Tree of Life Barcoding collective, Wellcome Sanger Institute Tree of Life programme, Wellcome Sanger Institute Scientific Operations: DNA Pipelines collective, Tree of Life Core Informatics collective, Darwin Tree of Life Consortium

**Affiliations:** 1 Amgueddfa Cymru, Cardiff, UK; 2 Marine Biological Association, Plymouth, Devon, UK

**Keywords:** Sthenelais limicola, segmented worm, genome sequence, chromosomal, Phyllodocida

## Abstract

We present a genome assembly from an individual *Sthenelais limicola* (the segmented worm; Annelida; Polychaeta; Phyllodocida; Sigalionidae). The genome sequence is 1,131 megabases in span. Most of the assembly is scaffolded into nine chromosomal pseudomolecules. The mitochondrial genome has also been assembled and is 16.7 kilobases in length.

## Species taxonomy

Eukaryota; Metazoa; Spiralia; Lophotrochozoa; Annelida; Polychaeta; Errantia; Phyllodocida; Sigalionidae; *Sthenelais*; *Sthenelais limicola* (Ehlers, 1864) (NCBI:txid1210413).

## Background

One of three *Sthenelais* species considered valid for UK and Irish waters, *Sthenelais limicola* is widely distributed throughout the northeast Atlantic, including the Mediterranean, and is also recorded from the northwest and southeast Atlantic ( Barnich & Fiege, 2003; Chambers & Muir, 1997). Found from littoral ( Parapar *et al*., 2015) to bathyal depths (1,550 m) ( Hartmann-Schröder, 1996), it inhabits sand and muddy substrates ( Parapar *et al.*, 2015).

*Sthenelais limicola* can be distinguished from other European species by the elytra having a smooth margin, bifurcate or notched, and a smooth surface with only a few microtubercles close to the point of attachment. Animals are generally colourless or white, with transparent elytra, often with a brownish patch on the posterior half, forming a V-shape or horseshoe appearance when combined with the appearance of the opposite elytron, and can reach up to 100 mm in size ( Hartmann-Schröder, 1996).

( Jumars *et al.*, 2015) classified all Sigalionidae as carnivores, although the method of hunting and prey are unknown for *S. limicola.* Little data is available on reproduction in Sigalionidae, but they are gonochoric as far as it is known ( Rouse *et al.,* 2022). It is neither under threat nor considered as a non-native species anywhere in the world.

The genome of the segmented worm, *Sthenelais limicola*, was sequenced as part of the Darwin Tree of Life Project, a collaborative effort to sequence all named eukaryotic species in the Atlantic Archipelago of Britain and Ireland.

## Genome sequence report

**Figure 1.**
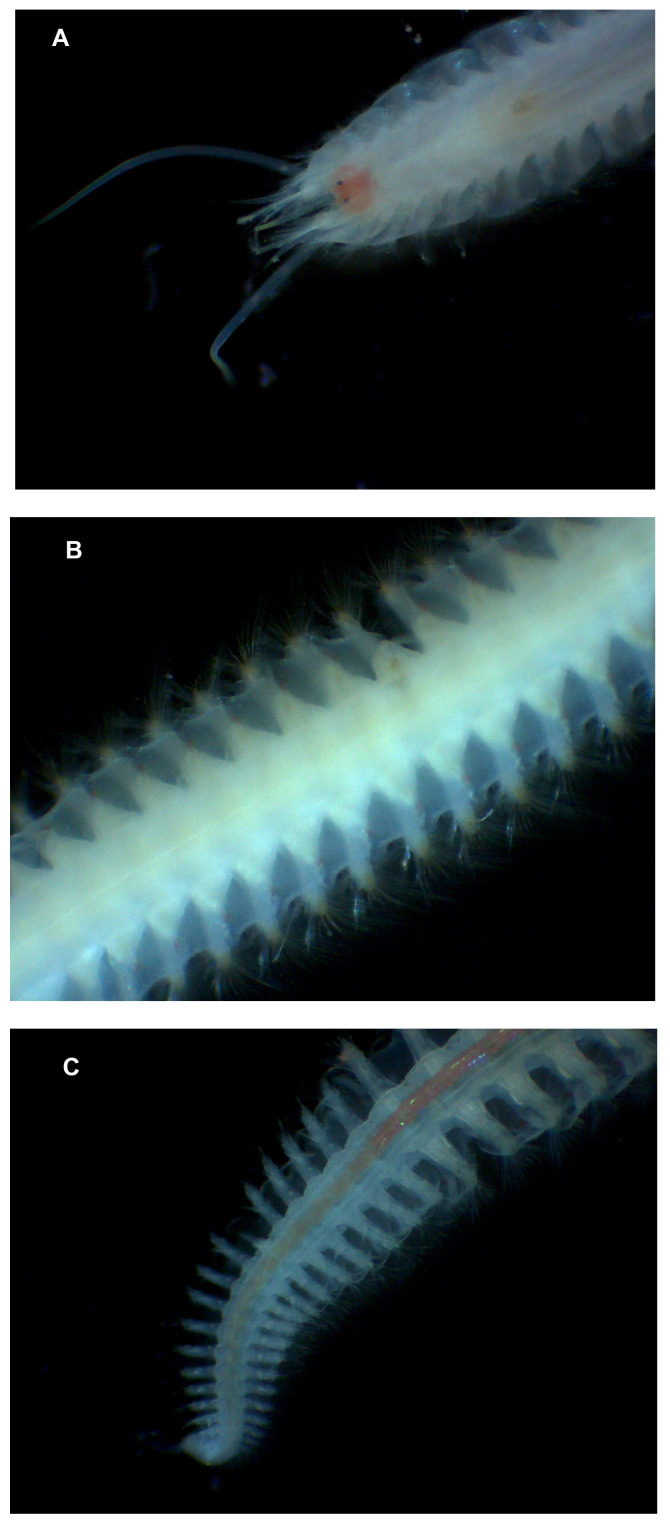
Photographs of the *Sthenelais limicola* (wpSthLimi1) specimen used for genome sequencing showing the dorsum of the head ( **A**), mid body ( **B**) and tail ( **C**).

The genome was sequenced from one *S. limicola* ( Figure 1) collected from East Breakwater, Plymouth Sound, UK (50.34, –4.14). A total of 45-fold coverage in Pacific Biosciences single-molecule HiFi long reads was generated. Primary assembly contigs were scaffolded with chromosome conformation Hi-C data. Manual assembly curation corrected 444 missing or mis-joins and removed 210 haplotypic duplications, reducing the assembly length by 3.71% and the scaffold number by 72.31%, and decreasing the scaffold N50 by 2.18%.

**Figure 2.**
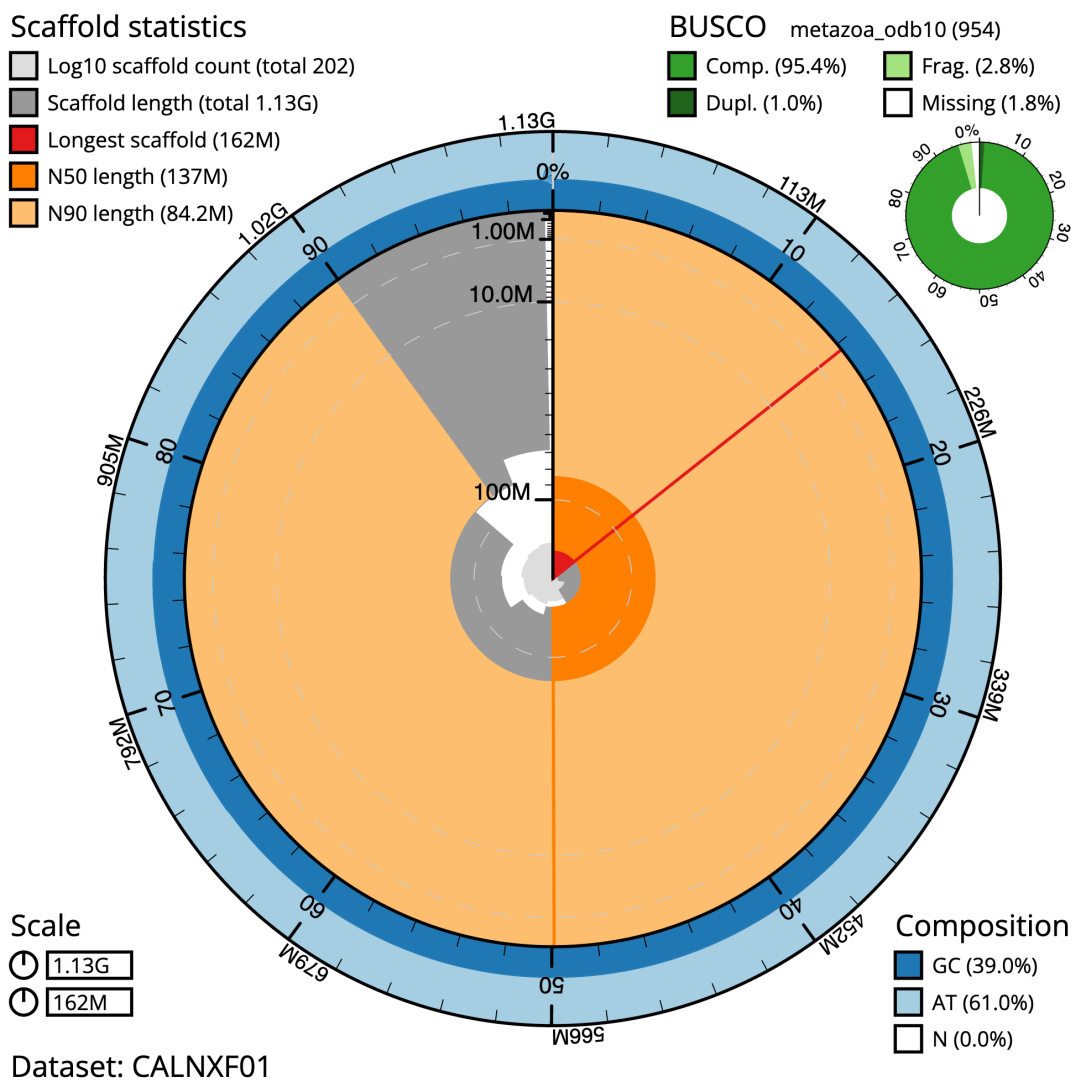
Genome assembly of *Sthenelais limicola*, wpSthLimi1.1: metrics. The BlobToolKit Snailplot shows N50 metrics and BUSCO gene completeness. The main plot is divided into 1,000 size-ordered bins around the circumference with each bin representing 0.1% of the 1,131,150,536 bp assembly. The distribution of scaffold lengths is shown in dark grey with the plot radius scaled to the longest scaffold present in the assembly (161,876,864 bp, shown in red). Orange and pale-orange arcs show the N50 and N90 scaffold lengths (136,979,486 and 84,233,480 bp), respectively. The pale grey spiral shows the cumulative scaffold count on a log scale with white scale lines showing successive orders of magnitude. The blue and pale-blue area around the outside of the plot shows the distribution of GC, AT and N percentages in the same bins as the inner plot. A summary of complete, fragmented, duplicated and missing BUSCO genes in the metazoa_odb10 set is shown in the top right. An interactive version of this figure is available at https://blobtoolkit.genomehubs.org/view/wpSthLimi1.1/dataset/CALNXF01/snail.

The final assembly has a total length of 1,131.1 Mb in 201 sequence scaffolds with a scaffold N50 of 137.0 Mb ( Table 1). Most (99.59%) of the assembly sequence was assigned to nine chromosomal-level scaffolds. Chromosome-scale scaffolds confirmed by the Hi-C data are named in order of size ( Figure 2– Figure 5; Table 2). The scaffold order and orientation is uncertain on chromosome 6 (0.39–8.86 Mb). Heterozygous inversion was observed on chromosome 7 (76.84–95.76 Mb). While not fully phased, the assembly deposited is of one haplotype. Contigs corresponding to the second haplotype have also been deposited. The assembly has a BUSCO v5.3.2 ( Manni *et al.*, 2021) completeness of 95.4% (single 94.3%, duplicated 1.0%) using the metazoa_odb10 reference set.

**Table 1.** Genome data for *Sthenelais limicola*, wpSthLimi1.1.

Project accession data		
Assembly identifier	wpSthLimi1.1	
Species	*Sthenelais limicola*	
Specimen	wpSthLimi1	
NCBI taxonomy ID	1210413	
BioProject	PRJEB51037	
BioSample ID	SAMEA8724794	
Isolate information	Mid-body tissue (DNA, RNA, Hi-C sequencing)	
Assembly metrics *		*Benchmark*
Consensus quality (QV)	59.9	≥ *50*
*k*-mer completeness	100	≥ *95%*
BUSCO **	C:95.4%[S:94.3%,D:1.0%], F:2.8%,M:1.8%,n:954	*C* ≥ *95%*
Percentage of assembly mapped to chromosomes	99.59%	≥ *95%*
Organelles	Mitochondrial genome assembled	*complete single alleles*
Raw data accessions		
PacificBiosciences SEQUEL II	ERR8978461–ERR8978463	
Hi-C Illumina	ERR8702825	
PolyA RNA-Seq Illumina	ERR10123685	
Genome assembly		
Assembly accession	GCA_942159475.1	
*Accession of alternate haplotype*	GCA_942183725.1	
Span (Mb)	1,131.1	
Number of contigs	1,310	
Contig N50 length (Mb)	10.2	
Number of scaffolds	201	
Scaffold N50 length (Mb)	137.0	
Longest scaffold (Mb)	161.9	

* Assembly metric benchmarks are adapted from column VGP-2020 of “Table 1: Proposed standards and metrics for defining genome assembly quality” from ( Rhie *et al.*, 2021). ** BUSCO scores based on the metazoa_odb10 BUSCO set using v5.3.2. C = complete [S = single copy, D = duplicated], F = fragmented, M = missing, n = number of orthologues in comparison. A full set of BUSCO scores is available at https://blobtoolkit.genomehubs.org/view/wpSthLimi1.1/dataset/CALNXF01/busco.

**Figure 3.**
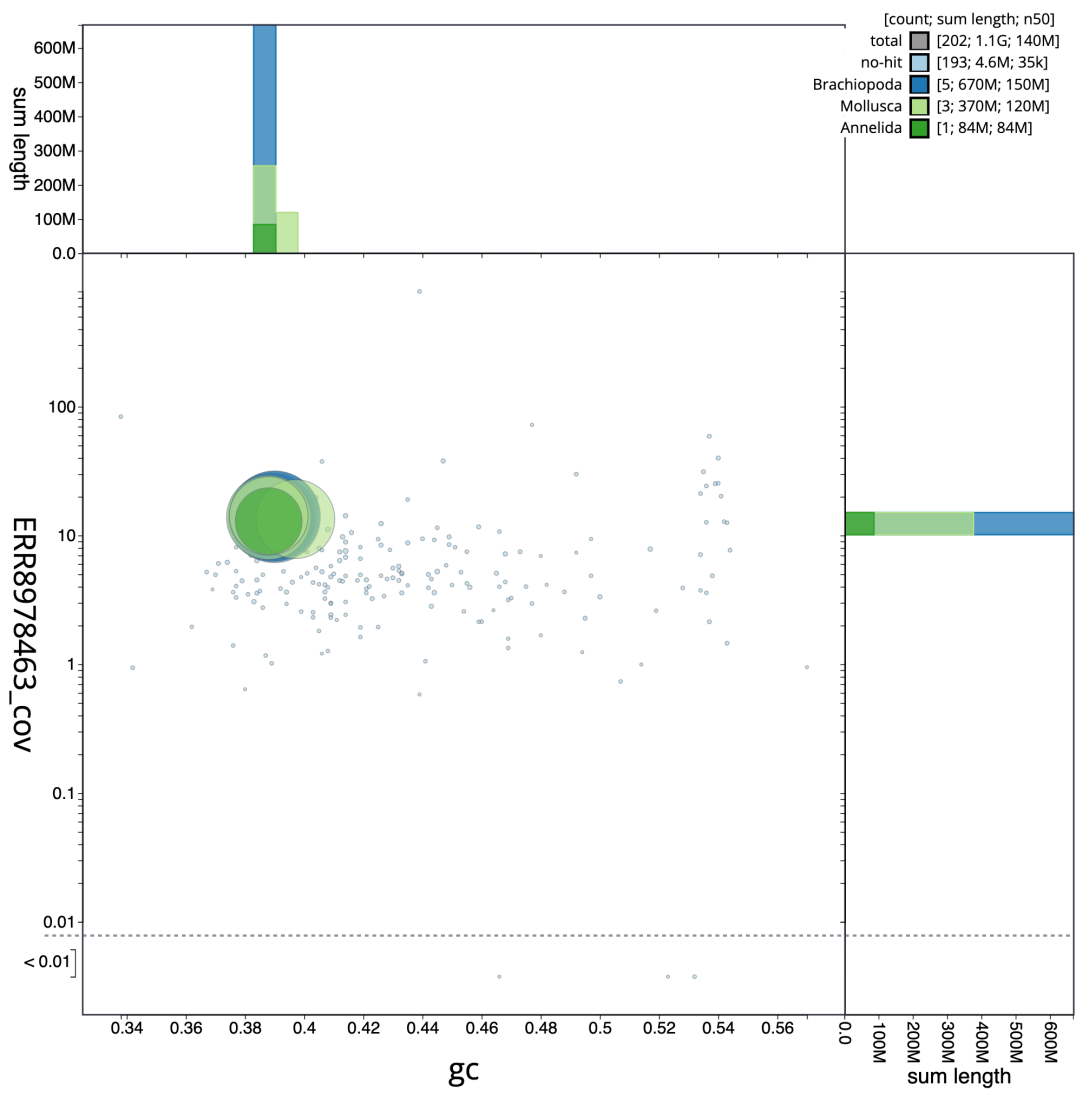
Genome assembly of *Sthenelais limicola*, wpSthLimi1.1: GC coverage. BlobToolKit GC-coverage plot. Scaffolds are coloured by phylum. Circles are sized in proportion to scaffold length. Histograms show the distribution of scaffold length sum along each axis. An interactive version of this figure is available at https://blobtoolkit.genomehubs.org/view/wpSthLimi1.1/dataset/CALNXF01/blob.

**Figure 4.**
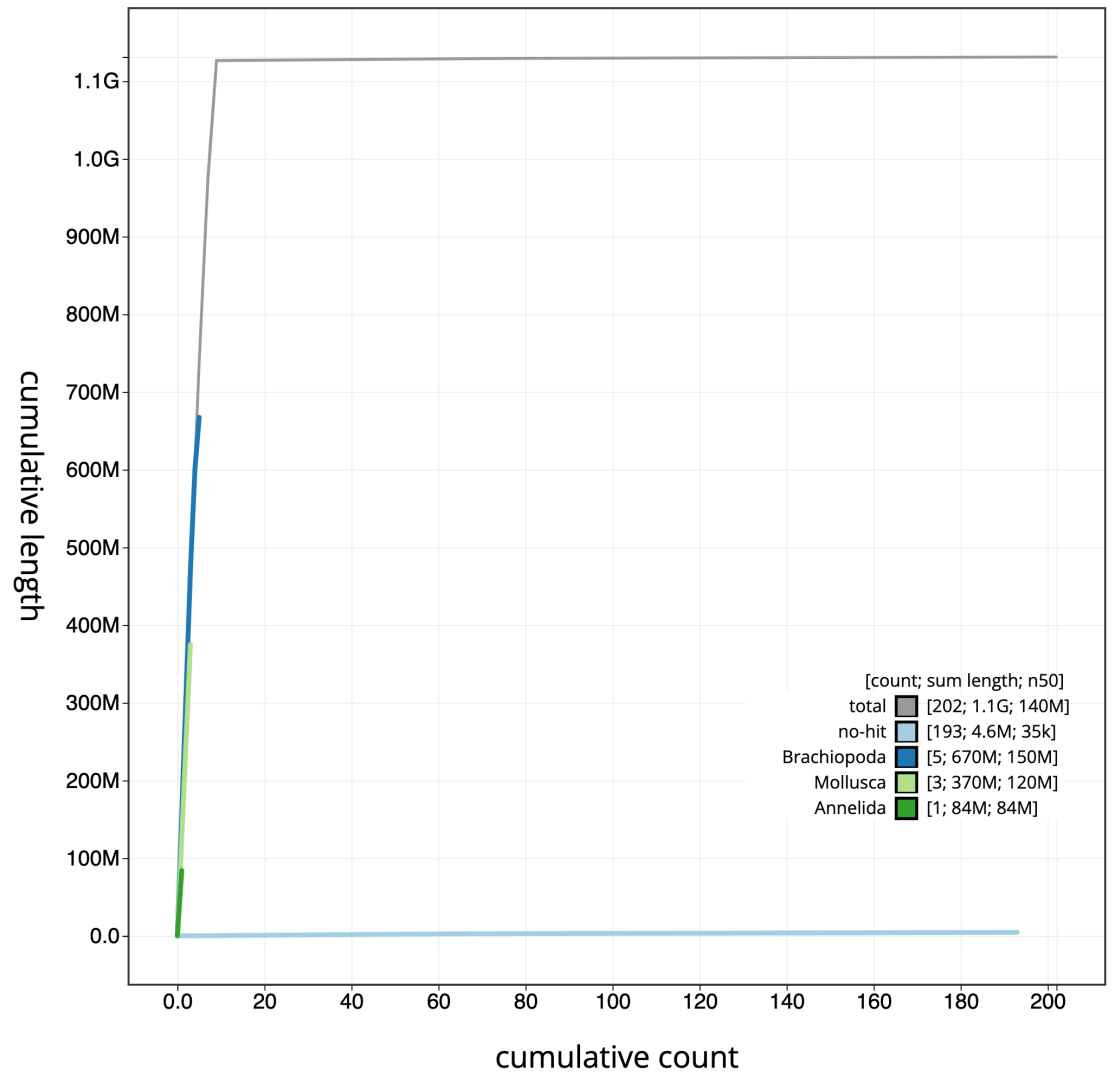
Genome assembly of *Sthenelais limicola*, wpSthLimi1.1: cumulative sequence. BlobToolKit cumulative sequence plot. The grey line shows cumulative length for all scaffolds. Coloured lines show cumulative lengths of scaffolds assigned to each phylum using the buscogenes taxrule. An interactive version of this figure is available at https://blobtoolkit.genomehubs.org/view/wpSthLimi1.1/dataset/CALNXF01/cumulative.

**Figure 5.**
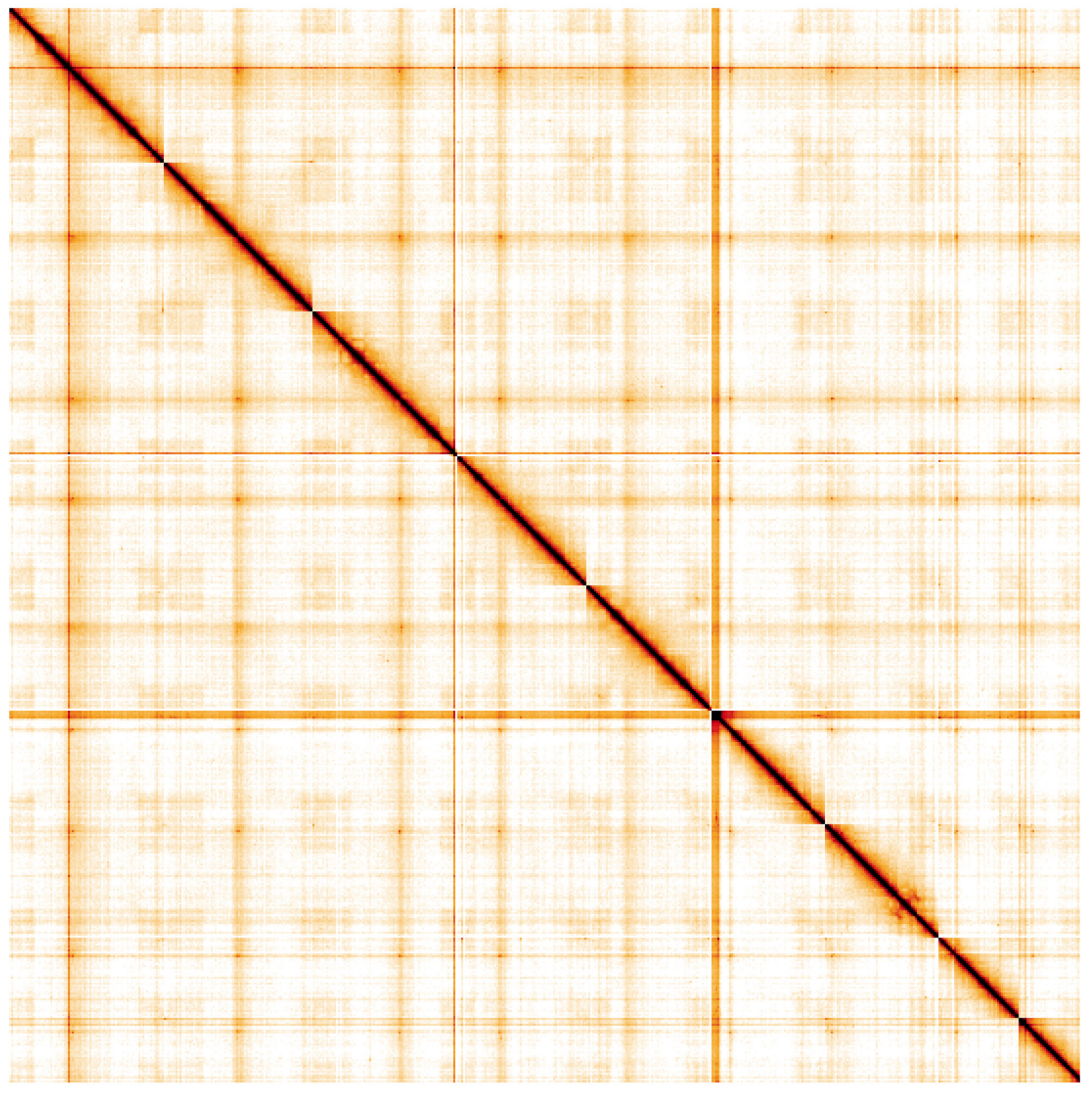
Genome assembly of *Sthenelais limicola*, wpSthLimi1.1: Hi-C contact map. Hi-C contact map of the wpSthLimi1.1 assembly, visualised using HiGlass. Chromosomes are shown in order of size from left to right and top to bottom. An interactive version of this figure may be viewed at https://genome-note-higlass.tol.sanger.ac.uk/l/?d=X-bn6mmVRHqGemal-zlDZA.

**Table 2.** Chromosomal pseudomolecules in the genome assembly of *Sthenelais limicola*, wpSthLimi1.

INSDC accession	Chromosome	Size (Mb)	GC%
OW804094.1	1	161.88	39
OW804095.1	2	156.73	39
OW804096.1	3	150.5	38.9
OW804097.1	4	136.98	38.8
OW804098.1	5	130.19	38.9
OW804099.1	6	119.44	39.7
OW804100.1	7	118.52	38.8
OW804101.1	8	84.23	38.8
OW804102.1	9	68.09	38.8
OW804103.1	MT	0.02	33.8

## Methods

### Sample acquisition and nucleic acid extraction

An individual *S. limicola* (wpSthLimi1; Figure 1) was collected from East Breakwater, Plymouth Sound, UK (latitude 50.34, longitude –4.14) by Teresa Darbyshire (National Museum Wales) and Mitchell Brennan and Sean McTierney (Marine Biological Association) and identified by Teresa Darbyshire. The sample was collected from muddy substrate using a grab sampler (MV Sepia) and preserved in liquid nitrogen.

DNA was extracted at the Tree of Life laboratory, Wellcome Sanger Institute. The wpSthLimi1 sample was weighed and dissected on dry ice with tissue set aside for Hi-C sequencing. Mid-body tissue was cryogenically disrupted to a fine powder using a Covaris cryoPREP Automated Dry Pulveriser, receiving multiple impacts. High molecular weight (HMW) DNA was extracted using the Qiagen MagAttract HMW DNA extraction kit. HMW DNA was sheared into an average fragment size of 12–20 kb in a Megaruptor 3 system with speed setting 30. Sheared DNA was purified by solid-phase reversible immobilisation using AMPure PB beads with a 1.8X ratio of beads to sample to remove the shorter fragments and concentrate the DNA sample. The concentration of the sheared and purified DNA was assessed using a Nanodrop spectrophotometer and Qubit Fluorometer and Qubit dsDNA High Sensitivity Assay kit. Fragment size distribution was evaluated by running the sample on the FemtoPulse system.

RNA was extracted from the mid-body tissue of wpSthLimi1 in the Tree of Life Laboratory at the WSI using TRIzol, according to the manufacturer’s instructions. RNA was then eluted in 50 μl RNAse-free water and its concentration assessed using a Nanodrop spectrophotometer and Qubit Fluorometer using the Qubit RNA Broad-Range (BR) Assay kit. Analysis of the integrity of the RNA was done using Agilent RNA 6000 Pico Kit and Eukaryotic Total RNA assay.

### Sequencing

Pacific Biosciences HiFi circular consensus and 10X Genomics read cloud DNA sequencing libraries were constructed according to the manufacturers’ instructions. Poly(A) RNA-Seq libraries were constructed using the NEB Ultra II RNA Library Prep kit. DNA and RNA sequencing was performed by the Scientific Operations core at the WSI on Pacific Biosciences SEQUEL II (HiFi) and Illumina NovaSeq 6000 (RNA-Seq) instruments. Hi-C data were also generated from wpSthLimi1 using the Arima v2 kit and sequenced on the Illumina NovaSeq 6000 instrument.

### Genome assembly

Assembly was carried out with Hifiasm ( Cheng *et al.*, 2021) and haplotypic duplication was identified and removed with purge_dups ( Guan *et al.*, 2020). The assembly was then scaffolded with Hi-C data ( Rao *et al.*, 2014) using YaHS ( Zhou *et al.*, 2022). The assembly was checked for contamination and corrected using the gEVAL system ( Chow *et al.*, 2016) as described previously ( Howe *et al.*, 2021). Manual curation was performed using gEVAL, HiGlass ( Kerpedjiev *et al.*, 2018) and Pretext ( Harry, 2022). The mitochondrial genome was assembled using MitoHiFi ( Uliano-Silva *et al.*, 2022), which performed annotation using MitoFinder ( Allio *et al.*, 2020). The genome was analysed and BUSCO scores generated within the BlobToolKit environment ( Challis *et al.*, 2020). Table 3 contains a list of all software tool versions used, where appropriate.

**Table 3.** Software tools and versions used.

Software tool	Version	Source
BlobToolKit	3.3.10	Challis *et al.*, 2020
gEVAL	N/A	Chow *et al.*, 2016
Hifiasm	0.16.1	Cheng *et al.*, 2021
HiGlass	1.11.6	Kerpedjiev *et al.*, 2018
MitoHiFi	1	Uliano-Silva *et al.*, 2022
PretextView	0.2	Harry, 2022
purge_dups	1.2.3	Guan *et al.*, 2020
YaHS	1.0	Zhou *et al.*, 2022

### Ethics/compliance issues

The materials that have contributed to this genome note have been supplied by a Darwin Tree of Life Partner. The submission of materials by a Darwin Tree of Life Partner is subject to the Darwin Tree of Life Project Sampling Code of Practice. By agreeing with and signing up to the Sampling Code of Practice, the Darwin Tree of Life Partner agrees they will meet the legal and ethical requirements and standards set out within this document in respect of all samples acquired for, and supplied to, the Darwin Tree of Life Project. Each transfer of samples is further undertaken according to a Research Collaboration Agreement or Material Transfer Agreement entered into by the Darwin Tree of Life Partner, Genome Research Limited (operating as the Wellcome Sanger Institute), and in some circumstances other Darwin Tree of Life collaborators.

## Data Availability

European Nucleotide Archive: *Sthenelais limicola* (a segmented worm). Accession number PRJEB51037; https://identifiers.org/ena.embl/PRJEB51037 ( Wellcome Sanger Institute, 2022). The genome sequence is released openly for reuse. The *Sthenelais limicola* genome sequencing initiative is part of the Darwin Tree of Life (DToL) project. All raw sequence data and the assembly have been deposited in INSDC databases. The genome will be annotated using available RNA-Seq data and presented through the Ensembl pipeline at the European Bioinformatics Institute. Raw data and assembly accession identifiers are reported in Table 1.

## Author information

Members of the Marine Biological Association Genome Acquisition Lab are listed here: https://doi.org/10.5281/zenodo.4783605.

Members of the Darwin Tree of Life Barcoding collective are listed here: https://doi.org/10.5281/zenodo.4893703.

Members of the Wellcome Sanger Institute Tree of Life programme are listed here: https://doi.org/10.5281/zenodo.4783585.

Members of Wellcome Sanger Institute Scientific Operations: DNA Pipelines collective are listed here: https://doi.org/10.5281/zenodo.4790455.

Members of the Tree of Life Core Informatics collective are listed here: https://doi.org/10.5281/zenodo.5013541.

Members of the Darwin Tree of Life Consortium are listed here: https://doi.org/10.5281/zenodo.4783558.
