# Supplementary material for: The genome sequence of the segmented worm, Sthenelais limicola (Ehlers, 1864)
Source: Wellcome Open Res. 2023 Jan 19;8:31. [Version 1] doi: 10.12688/wellcomeopenres.18856.1 (PMC10352628; doi:10.12688/wellcomeopenres.18856.1)
Supplement: Peer review history

# Peer review: Reviewer response for version 1

**DOI:** 10.21956/wellcomeopenres.20909.r61558

**Published:** 2023-07-17

**Authors:** Erich Schwarz

**Affiliations:** 1 Cornell University, Ithaca, New York, USA

This report concisely but clearly describes the chromosomally scaffolded genome assembly of a segmented worm first identified in 1864 but not given serious genomic analysis until now. The text is well-written with explicit, well-referenced, and versioned software tools in its methods. The data are publicly available on GenBank for further work.

 Notably, the authors did not do predictions of protein-coding genes, but did provide RNA-seq data that would support such prediction by other, interested parties.

 Although the authors used automatic defaults for initial assembly of HiFi reads by HiFiAsm and removing excess heterozygous alleles with purge_dup, but put substantial manual effort into making the most accurate Hi-C chromosomal scaffolding that they could. The methods they describe for careful manual correction of Hi-C scaffolding after initial use of YaHS, a recently devised Hi-C scaffolder that may be superior to 3d-dna.

 Overall, this report provides the first serious genomic resources for this category of segmented worms, and these resources are well-constructed enough to support future work of high quality.

Are sufficient details of methods and materials provided to allow replication by others?

Yes

Is the rationale for creating the dataset(s) clearly described?

Yes

Are the datasets clearly presented in a useable and accessible format?

Yes

Are the protocols appropriate and is the work technically sound?

Yes

Reviewer Expertise:

Assembly, annotation, and biological analysis of nematode genomes: this includes both free-living Caenorhabditis nematodes and parasitic nematodes (e.g., hookworms).

I confirm that I have read this submission and believe that I have an appropriate level of expertise to confirm that it is of an acceptable scientific standard.

# Peer review: Reviewer response for version 1

**DOI:** 10.21956/wellcomeopenres.20909.r57955

**Published:** 2023-07-10

**Authors:** Torsten H Struck

**Affiliations:** 1 University of Oslo, Oslo, Oslo, Norway

The paper by Darbyshire et al presents the nuclear and mitochondrial genome of an annelid scale worm at the reference level and to my knowledge it is the first for the annelid group Aphroditiformia. It is all in all very well described and has all essential statistics. However, I would like to make a few suggestions, which will help to increase repeatability of the study and all researchers not so familiar with genomics research to use this study as their starting point for conducting their genome projects. Especially, annelid researcher might use such a paper as a starting point. At present, this however not possible as a lot information is lacking describing the bioinformatic parts of this project.

 In the description of the genome assembly are the settings for the different programs not given. Moreover, also any custom-made scripts used for the analysis are not mentioned. However, given the different data sources it has to be assumed that these scripts are present. The settings and scripts should be made publicly available to allow repetition of the analyses as the authors did.

 Additionally, while the lab methods have been described in sufficient detail, several details on the bioinformatic analyses are missing. Which programs have been used for the mapping, blast/diamond and BUSCO analyses needed for blobtools analysis? BlobTools does not do it by itself. Which programs were used to assess the different quality parameters such as N50, kmer completeness, consensus quality and so forth? Also for the HiC, analyses crucial steps in preparation of the data for YAHS analysis are not described. For some one, new to the field it will very challenging to repeat the analyses easily. If these methods have been published in a different paper the authors should refer to this paper and indicate any deviations from it.

 Other points to consider are:

 The members of the barcoding team are listed as authors. However, the barcoding approach is not described in the paper at all and also none of its result. Did the barcode confirm the morphological species? This is to be assumed as the policy of DToL is to sequence only species with a matching barcode. Nonetheless a statement should be made in the paper. Moreover, where is the barcode deposited? No accession number has been provided.

 Similarly, the generation of mitochondrial genomes is mentioned in the methods, but only described as "complete single alleles" in Table 1. It is not obvious what this description means? Is the full genome retrieved with different somatic, intraspecific haplotypes or is the genome recovered in several pieces? The accession number is provided for only one entire, non-annotated mitochondrial genome. Hence, what are the other allels?

 Additionally, a statement concerning the fulfillment of the EBP standards for chromosome-level assemblies as of today would be nice. This could ne the EBP code of quality, which in this case would be 7.C.Q60.

 Finally, "Polychaeta" is a paraphyletic group and hence the name "Polychaeta" should not be used. If the authors want to use the term polychaetes, they should use it as polychaetes and not "Polychaeta". The latter would indicate that it is a proper name and monophyletic group. If the authors want to display the NCBI taxonomy here, the authors should name the section NCBI taxonomy and not Species taxonomy as in this case it is not the latter given all recent phylogenetic studies on Annelida showing the paraphyly of polychaetes.

Are sufficient details of methods and materials provided to allow replication by others?

Partly

Is the rationale for creating the dataset(s) clearly described?

Yes

Are the datasets clearly presented in a useable and accessible format?

Partly

Are the protocols appropriate and is the work technically sound?

Yes

Reviewer Expertise:

Evolutionary Genomics; Phylogenetics; Systematics and Taxonomy of Annelida

I confirm that I have read this submission and believe that I have an appropriate level of expertise to confirm that it is of an acceptable scientific standard, however I have significant reservations, as outlined above.

# Peer review: Reviewer response for version 1

**DOI:** 10.21956/wellcomeopenres.20909.r54382

**Published:** 2023-02-06

**Authors:** Joseph Ryan

**Affiliations:** 1 Whitney Laboratory for Marine Bioscience, University of Florida, St Augustine, FL, USA

This article describes the sequencing and assembly of the *Sthenelais limicola *genome. The description of the methods are sound and the analyses are repeatable. The quality metrics are stellar. The manuscript is clearly written and the figures and figure legends are easy to comprehend. 

 One minor point is the description of the tissue that was sequenced for gDNA and RNA sequencing could be better described. This tissue was described as " *mid-body tissue*." I am guessing that this was a cross section of the animal at one of the middle segments, but this could refer to ectoderm only, gut only, etc. If possible, it would help to clarify (a bonus would be the segment number(s)).

Are sufficient details of methods and materials provided to allow replication by others?

Yes

Is the rationale for creating the dataset(s) clearly described?

Yes

Are the datasets clearly presented in a useable and accessible format?

Yes

Are the protocols appropriate and is the work technically sound?

Yes

Reviewer Expertise:

evolutionary genomics

I confirm that I have read this submission and believe that I have an appropriate level of expertise to confirm that it is of an acceptable scientific standard.
